# Supplementary material for: Changing face of socio-economic vulnerability and COVID-19: An analysis of country wealth during the first two years of the pandemic
Source: PLoS One. 2023 Aug 28;18(8):e0290529. doi: 10.1371/journal.pone.0290529 (PMC10461814; doi:10.1371/journal.pone.0290529)
Supplement: Attachment — Submitted filename: Reviewers Comments.pdf [file pone.0290529.s002.pdf]

The authors conducted an ecological study of global scope with a consecutive application of cross-sectional analysis during first two pandemic years of COVID 19. An analysis of country wealth was conducted. This study is important as it shows the “changing face” of the pandemic, mostly affecting high income countries at first and later the low income countries.

However, the following are my comments.

In lines 10-11…The sentence “And on the other hand to find out whether the statistical production capacity of the country is a confounding variable for COVID-19 cases and deaths” needs rephrasing.

In lines 11-12 the authors wrote that “To achieve the objectives, an ecological study of global scope is proposed”. This sentence needs rephrasing as the study has already been conducted.

In line 12-14 the sentence “Whose analytical strategy is the consecutive application of cross-sectional analysis…” should be rephrased.

The authors conclude that poor statistical production capacity of poorer countries may be masking the real incidence of the disease. However, the definition of poor statistical production and was not clearly explained. In addition, I would suggest the use of “low income countries” rather than “poorer countries” in the statement.
