# Supplementary material for: Changing face of socio-economic vulnerability and COVID-19: An analysis of country wealth during the first two years of the pandemic
Source: PLoS One. 2023 Aug 28;18(8):e0290529. doi: 10.1371/journal.pone.0290529 (PMC10461814; doi:10.1371/journal.pone.0290529)
Supplement: Attachment — Submitted filename: Comments.docx [file pone.0290529.s001.docx]

It is my pleasure to review your paper. Under the hypothesis of the dynamic and changing over time, the main objective of your paper was to determine the relationship between the country's wealth and the incidence of COVID-19. However, I have a few comments as follows;

1. There is no adequate information on the methods. The authors should indicate how data were collected, the sampling technique, the setting of your study, etc.
2. Equations 1 – 3 on pages 7 and 8, should be included in the methods instead of being presented in the results.
3. The conclusion should be summarized.
4. The authors didn’t indicate any ethical considerations. It’s important to be indicated in the study since it involved studying the incidence of COVID-19 that happened to people.
5. The list of abbreviations is missing in the main document.
