# Supplementary material for: Changing face of socio-economic vulnerability and COVID-19: An analysis of country wealth during the first two years of the pandemic
Source: PLoS One. 2023 Aug 28;18(8):e0290529. doi: 10.1371/journal.pone.0290529 (PMC10461814; doi:10.1371/journal.pone.0290529)
Supplement: Attachment — Submitted filename: Response to reviewers.docx [file pone.0290529.s003.docx]

Dear Professors,

We would like to thank the reviewers to help us to improve the quality of this manuscript with their helpful comments and constructive suggestions, we have introduced several clarifications and comments in the manuscript that we consider that can help to their better understanding.

We look forward to your response,

The authors

Response. Reviewer 1

1. **There is no adequate information on the methods. The authors should indicate how data were collected, the sampling technique, the setting of your study, etc.**

- In the methods section, a sub-section called measures has been included where information on the units of analysis and measures has been included (Pages 6-10).

2. **Equations 1 – 3 on pages 7 and 8, should be included in the methods instead of being presented in the results.**

- The equations have been included in the methods section (Page 6) along with a brief explanation of the analytical strategy (Page 5, lines 20-25; Page 6, lines 1-2).

3. **The authors didn’t indicate any ethical considerations. It’s important to be indicated in the study since it involved studying the incidence of COVID-19 that happened to people.**

- Since this is a macro analysis at the national level on data from secondary data sources, the work does not jeopardize the privacy of the participants. All data used in this analysis are publicly available and can be found at: : <https://github.com/vicperez/Changing-Face-of-Socio-Economic-Vulnerability-and-COVID-19> (Page 6; lines 26-30).

4. **The conclusion should be summarized.**

- A summary of the paper's findings and contributions is included at the end of the article (Page 16, lines 7-21).

5. **The list of abbreviations is missing in the main document.**

- A list of abbreviations has been included at the beginning of the main manuscript (page 2, line 6).

Response. Reviewer 2

**In lines 10-11…The sentence “And on the other hand to find out whether the statistical production capacity of the country is a confounding variable for COVID-19 cases and deaths” needs rephrasing**

The sentence has been rephrased as: “And on the other hand, to find out if the country's statistical production capacity is masking the real incidence of the COVID-19 pandemic” (Page 2, lines 13-14).

**In lines 11-12 the authors wrote that “To achieve the objectives, an ecological study of global scope is proposed”. This sentence needs rephrasing as the study has already been conducted.**

The sentence has been rephrased as: In order to achieve this objective, an ecological study has been designed at international level with the countries established as study units” (Page 2, lines 14-16)

**In line 12-14 the sentence “Whose analytical strategy is the consecutive application of crosssectional analysis…” should be rephrased.**

The sentence has been rephrased as: “The analytical strategy utilized involves the consecutive application of cross-sectional analysis, specifically employing multivariate linear regression on a daily basis throughout the first two years of the pandemic (from 03/14/2020 to 03/28/2022)” (Page 2, lines 16-18).

**The authors conclude that poor statistical production capacity of poorer countries may be masking the real incidence of the disease. However, the definition of poor statistical production and was not clearly explained. In addition, I would suggest the use of “low income countries” rather than “poorer countries” in the statement**

- Statistical production capacity has been defined in the subsection Measures of the section Method (Pages 6-10)
- Poor countries have been replaced by low income countries (Page 3, line 5; Page 15, line 19; Page 16, lines 12-13),
